# Hospital and Institutional News

**Published:** 1918-02-16

**Authors:** 


### HOSPITAL AND INSTITUTIONAL NEWS.

### MUNIFICENT BEQUESTS BY A HOSPITAL CHAIRMAN.

The total sum bequeathed for charitable and educational purposes by the late Sir Edward Wood, J.P. (particulars of whose life and work appeared in The Hospital of November 5, 1910, and September 29, 1917), is approximately £50,000. To the Leicester Royal Infirmary, of which institution he was chairman, and the founder of the reconstruction scheme, he bequeathed £1,000 each for the endowment of two beds to the memory of himself and his wife, and £1,000 to the erection of a new pathological department, this being a contribution which he promised in his lifetime. Subject to certain life interests, he also bequeathed out of his residuary estate £4,000 for the endowment of four beds to the memory of members of his family, and £1,000 for the establishment of a medical library in the institution. To the Children's Hospital in connection with the infirmary he bequeathed £500 for the endowment of a cot, and, subject to certain interests, £1,500 from his residuary estate for the endowment of three cots to the memory of members of his family. The total bequests to these institutions are thus £10,000. Other bequests included £2,000 to the Wyggeston Hospital Schools at Leicester; for the foundation of an "Edward Wood" scholarship tenable at one or other of the universities approved by the governors of the school, and renewable for a period not exceeding four years; £1,000 to the Leicester Charity Organisation Society; £500 and, subject to certain interests, a further £500 from his residuary estate to the Leicester District Nursing Association and Home; £100 each to the Leicester Maternity Hospital, the Infant Orphan Asylum, Leicester, the Leicester branch of the Surgical Aid Society, the Leicester Branch Association for the Blind, the Wycliffe Association for the Blind, and the Leicester Homœopathic Hospital; £1,000 to the Leicester Guild of the Crippled, £1,000 to Dr. Barnardo's Homes, and £100 to the Military Training School in connection therewith. He further bequeathed to his trustees £5,000, and, subject to certain life interests, a further sum of £19,000 to form an "Edward Wood Bequest Fund," to be administered by a board of management in the granting, under certain conditions, of annuities of £20 per annum to gentlewomen; and a further sum of £5,000 to his trustees for investment for the "Edward Wood Poor Widows' House Rent Assistance Fund."

### MR. COLES' SUCCESSOR AT CAMBRIDGE.

Mr. W. H. Head has been appointed to succeed Mr. Richard J. Coles as secretary superintendent at Addenbrooke's Hospital. Mr. Head is a Fellow of the Chartered Institute of Secretaries and has spent twenty-four years in hospital work. For eleven years he was assistant secretary of the Cheltenham General Hospital, where he has been the secretary since 1905. Mr. Head's testimonials were regarded at Cambridge to be of the highest, and his work at Cheltenham as excellent. We wish Mr. Head success in his new field of labour, which his predecessor made so productive and fruitful in results.

### THE STAFF RECORD AT HALIFAX ROYAL INFIRMARY.

The art of attaching loyal and therefore long service is one of the proofs of good administration, and the record of the Halifax Royal Infirmary in this regard is noteworthy. Mr. Oates Webster, the secretary, has held the post for thirty-one years. His wife, a former matron, who severed her connection with the institution six years ago, was in its service for a similar period. Among the sisters, Sister Shelvock has spent twenty-four years at the hospital from her days as probationer till her promotion to acting-matron. Sister Fox will soon have been there for twenty years. For this period Sister Shelvock's predecessor in the post of acting-matron, Miss Aspinall, worked for the hospital. The chief porter has been at the hospital for a quarter of a century, and the head-laundress started work in 1886. What other hospital can improve on this?

### HOSPITAL BUILDINGS AFTER THE WAR.

The Yeovil and District Hospital, which now serves an increased population of industrial workers, is hoping to obtain permission from the Ministry of Munitions to undertake a building scheme. Apart from the need, various arguments in favour of an immediate start have a general interest. Mr. W. McMillan has declared his opinion that the opportunity will not be better after the war. The town at the moment is prosperous; the cost of building has much increased, but in Mr. McMillan's opinion the high price of building will last at least for ten years. Moreover, he was sure that an appeal would be better supported now than later, and, as a memorial to the fallen, a new hospital could not fail to be popular. To fill up the form of application demanded by the Ministry of Munitions plans will have to be drawn in detail. That is certainly worth doing, but while the accident ward is closed, and notwithstanding the risks which attend the munition workers, might not the gap be filled and the appeal encouraged by the erection of the temporary buildings which have proved so successful elsewhere?

### DOCTORS' FEES FOR ARMY PATIENTS.

In his recent speech to the governors the chairman of the Royal Infirmary, Dumfries, the Rev. J. Montgomery Campbell, referred to the present position of the hospitals in respect of military patients. After remarking that his hospital was affiliated to the British Hospitals Association, Mr. Campbell referred to the agreement between it and the Ministry of Pensions, under which the sum of 35s. a week was suggested as the payment for each patient. This, he went on, could not be regarded as excessive if one considered that the actual cost per patient per day was 4s. 5¾d., exclusive of rent for buildings or fees for medical treatment. This raised an important question for the doctors, who, while ready to minister to the sick poor, were now being asked to attend patients for whom the institution was receiving payment. Under the circumstances he thought that the doctors had a just claim. At present a percentage was paid to the surgeon who attended those cases of non-pulmonary tuberculosis which were paid for by the Insurance Committee, and he thought that this was only fair. This speech, no doubt, will find supporters, and the situation which evoked it has an interest. For we see on looking back that no sooner did the voluntary hospitals begin to accept payment for the treatment of cases than two secondary results followed. The first was the demand for fees on the part of the medical staff. The second was that fees for treatment in hospitals are now being computed on the basis not of the cost of treatment only, but of the cost of other things as well, which may be conveniently summarised as rent and interest. Is the time coming when the voluntary hospitals will expect to make a profit on the cases for which they are paid on the ground, of course, that such a profit enables them to treat certain other patients free? This, at all events, would be the position if fees were assessed to cover, beyond the cost of maintenance, rent on buildings and medical fees. When the military patients cease to arrive in new batches the more chronic cases will remain to perpetuate a precedent after the war, and it may easily be applied to other patients—like those sent by Insurance Committees—who are supported by the public funds.

### THE HOSPITAL STANDARD IN LEEDS.

Because of the waiting list and the pressure in emergencies Leeds Maternity Hospital is appealing for the endowment of beds, and to cope with the work the income should be raised at least to £10,000, or almost double the existing figure. Indeed, before very long larger premises will become essential, and the hospital's work for the university, by which it is recognised, and for the corporation, which has made of it the centre of a maternal and infant welfare scheme for the city, should enable it to organise subscriptions from the different classes which it serves to a degree which has been difficult in the past. Soldiers' wives and war workers are, of course, among the patients. The hospital standard at Leeds has been raised of recent years, and it is generally found that where the standard is raised there, too, is created the desire and the money to impose it on all the hospitals of a city.

### THE FIRST TWO 1917 HOSPITAL REPORTS.

We always look forward at this time of the year with interest to see which will be the first hospital to send for notice a copy of the accounts for the previous year. The honour on this occasion belongs to the Royal West Sussex Hospital, Chichester, whose preliminary statement reached us in January. Technically this hospital is not the winner, as the statement of income and expenditure has not been audited, and is therefore subject to alteration and possibly incomplete. The year's accounts show a balance on the wrong side of £844, and it is noted that the expenditure includes the cost of maintenance of soldiers, towards which £1,456 was paid by the War Office, a sum which the committee considers barely covers the cost. The extra grant of 9d. per day per man arranged by the British Hospitals Association will no doubt remove any uncertainty as to whether the hospital is out of pocket. The first report with audited accounts is that of the Dewsbury and District General Infirmary, but unfortunately the financial statement is not in accordance with the Uniform System of Accounts. The accounts are so near that system, however, that a small amount of alteration and but little attention on the part of Mr. Midgley, the treasurer, will put them right. We have still to receive an orthodox and audited set of accounts for 1917. Which hospital will prove the winner in the race?

### SURRENDER WEEK.

A number of metropolitan and provincial hospitals are benefiting by the opportunity that the Food Controller has given to those who are in possession of unreasonable stores of food. It is amusing to study the various methods of secretaries in the business of coming into touch with the owners of what is just now a dangerous form of property. Some are tactful and clever, some are tactless and maladroit. We like particularly Mr. Carroll's appeal on behalf of All Saints' Hospital, that any "accidental surplus" of food should be sent to Vauxhall Bridge Road. This, we are informed, brought not only grist but other things to the mill, and one clever idea has been followed up by another, for we are to-day informed that the hospital will be glad to receive donations and subscriptions from those who feel that they have been eating more than their fair share of what food is procurable. The matron of the National Hospital for the Paralysed was first in the field, and in a quiet way has for some weeks been acknowledging the receipt of anonymous presents of food, and asking for more, which we believe have been given in an interestingly varied form, the contents of parcels ranging from a pot of marmalade to six dozen packets of cornflour. Plagiarism in hospital appeals is not uncommon, but we fancy that advertisements brutally headed "Food Hoarding" are not likely to touch the hearts of those who, in many cases quite innocently, are in possession of a too ample supply of eatables.

### A SUGGESTION TO HOSPITAL COLLECTORS.

To endow beds many devices have been tried, of which the memorial bed has probably been the most successful. With the present attempt to organise sectional subscriptions from each separate class which a hospital serves district beds (for classes and districts are often associated) are being revived. In the case of those districts which send many more patients than Treasury notes to their local hospital, collectors would probably find it easier to induce subscribers to give to "our bed"

than merely to subscribe to a more or less distant hospital. The idea is not new, but an idea has better luck at some seasons than at others. Now that the difficulty of maintenance is really more severe than ever with the recognition that the scandal of long waiting-lists cannot be tolerated indefinitely, any suggestion with a chance of success is welcome. One enthusiast has succeeded in collecting a thousand pounds to endow a bed, in the interests of his district, at a big hospital in the West, and we hope that other districts elsewhere which have been deaf to a collector may respond to an appeal to have a bed of their own.

### MUNICIPAL CONSULTATIONS.

The Public Health Committee of the West Riding County Council has resolved on a new departure. A consultant branch has been established in connection with the Scalebor Park institution. The committee felt that in so specialised a subject as mental illness the services of the medical superintendent should be available for any medical practitioner who wished for a second opinion or any patient who desired advice. It is known that many have already taken advantage of the experience of the medical staffs at Scalebor Park and other institutions under the County Council, and so the committee has now decided to organise a consultant department. In order that the feeling of coming to such an institution should be as far as possible avoided, it has been agreed that the department shall ultimately be established at the "Highlands," which is near a railway station and convenient of access. Appointments can be arranged at any time through the medical practitioner in attendance. The Health Committee hopes that by this means many cases of mental illness may be brought under treatment at an early period and the development of more serious symptoms prevented. The fees to be charged will be by arrangement with the medical superintendent, but there will be nothing to prevent the medical superintendent treating free of charge any patient whose circumstances do not permit of the payment of a fee.

### DISCHARGED EPILEPTIC SOLDIERS.

Sixteen discharged epileptic soldiers have been admitted to the Monyhull Colony in pursuance of an agreement between the Birmingham Guardians and the Ministry of Pensions. The men are being trained in various kinds of farm and gardening work under the personal supervision of the farm bailiff. Recently the Parliamentary Secretary of the Ministry of Pensions, Sir Arthur Boscawen, and Dr. Bond visited the home set apart for these patients, and expressed their entire satisfaction with the arrangements that had been made for their care and control, and particularly noticed the contented manner in which the men had settled down.

### THE VALUE OF MEDICAL EXPERT EVIDENCE.

The value of expert medical evidence was again demonstrated at the Lambeth County Court in a claim for compensation under the Workmen's Compensation Act. The applicant, who was making mop-poles out of imported timber, alleged that whilst he was at work his plane came in contact with an explosive bullet embedded in the wood, and that it exploded, a bullet or some other substance penetrating the palm of the left hand and coming out at the back. His story, however, was not accepted after Judge Hodges had heard the medical evidence. After the alleged accident the man was taken to Guy's Hospital, where he was seen by Dr. Arthur Bulleigh, the house surgeon, and an $x$-ray picture was taken. Dr. Bulleigh, who was supported in his contention by Dr. G. Stebbings and Dr. McColl, assistant medical officers at Lambeth Infirmary, where the man was afterwards treated, maintained that the bullet entered the hand from the back, where the wound was smaller than in the palm, where it was lacerated. Three doctors held that the applicant's account of the accident was impossible and inconceivable. The judge naturally was guided by the expert evidence, and gave judgment for the man's employers.

### DENTAL TREATMENT FOR MOTHERS.

Dr. J. Francis Taylor, the medical officer of health for Leyton, reports that the infant welfare and ante-natal clinics are making rapid strides in popularity. Attendances and results are most satisfactory, but he suggests that in order to develop their usefulness an extension is advisable in the direction of providing dental treatment for actual and prospective mothers. It is found that the health of the mothers is seriously prejudiced by the presence of decaying teeth, which act as a septic focus from which toxins are absorbed into the blood, and these have been proved to affect injuriously the unborn infant and also the milk of the nursing mother. There is already a dental clinic for school children with a dentist and nurse conducting the work, and he suggests that arrangements should be made for two sessions at the ante-natal clinic. Dr. Taylor would administer the necessary anæsthetics, as he does in the case of the school children. The total expenditure, excepting a small sum for fillings, would not exceed £125 per annum, one-half of which would be payable by the Local Government Board. Dr. Taylor predicts that incalculable benefit would be conferred on the mothers and infants by this modest outlay. The Health Committee has agreed to this proposal.

### BABY CARE IN PLYMOUTH.

Dr. O. Hall, the medical officer of health for Plymouth, is able to announce in his annual report that the infantile death-rate last year was the lowest on record in the borough—viz. 90.6 per 1,000, as compared with 119.3 the previous year. This marked reduction is a result, no doubt, of the child-welfare efforts, which are bearing fruit almost everywhere. The good results anticipated from the compulsory notification of measles, says Dr. Hall, have not so far been attained. Last year 2,143 cases of measles were notified, with forty-nine deaths, as against 1,655 notifications in the previous year and 116 deaths. Dr Hall has found cinema theatres a source of anxiety. "Packed with people of all ages, frequently overheated, and often inadequately ventilated, they furnish in condensed form conditions which must depress vitality, predispose to chills, and facilitate the spread of infectious disease." Continuous supervision has been necessary, adds Dr. Hall, to maintain the required standard of sanitation. In order to increase the practical efficiency of the midwives of Plymouth Dr. Hall has embarked on a course of free lectures, with demonstrations, an invitation being specially extended to women who were in practice prior to the passing of the Midwives Act, 1902.

### ILLEGITIMACY AND DEATH.

There is something suggestive of an Armenian massacre associated with a report of the Hammersmith Borough Council's Health Committee. Attention was drawn particularly to a paragraph in the annual report of the medical officer of health, dealing with the deaths of illegitimate children, and the committee says that of the total number—namely, 222—of children who died in Hammersmith before attaining the age of one year, 42 were illegitimate. The mortality amongst the illegitimate children was at the astonishing rate of 538 deaths per thousand, which compared with a mortality of only 69 per thousand amongst legitimate children. The committee declares that it is strongly of opinion that the high mortality amongst the illegitimates under one year is largely due to the lack of proper supervision. At present what supervision exists is carried out by the London County Council, which has authority for licensing persons to receive infants for reward. If, urges the committee, this authority were vested in the Borough Council, with its more intimate knowledge of local conditions, there is every reason to believe that the mortality would be considerably reduced.

### POISONOUS TREES.

It is reported that two of the guests at a Red Cross dinner at Budapest became ill through smoking "tobacco" made of beech leaves. If true, this story is surprising. In Evelyn's time the leaves of the beech were considered to be "wholesome for the gums and teeth" when chewed, and the buds, hardened and dried, were used in the seventeenth century as toothpicks. If the "well-furnish'd and glistering leaves" of which *Sylva* speaks have now become poisonous, it is probably because of the treatment they receive on the way to being fitted (more or less) for smoking. Simultaneously with this account of the dire results of experiments with strange smoking mixtures, comes a case in the Court of Appeal which turned upon the poisoning of some cattle from eating the branches of yew-trees. That cattle have often died after browsing upon the yew is undoubted; on the other hand, they often experience no ill effects. The old theory was that the branches were harmful only when they were newly sprouting. Aubrey tells of two ladies who perished from drinking a decoction of pounded yew under the impression that it would cure an obstinate disease.

### REFLECTIONS ON PEACE.

The gazette of the 3rd London General Hospital, Wandsworth, generally salts for its humour by something more ambitious than humour in ordinary hands can claim to be. Miss H. M. Nightingale more than once has come to the rescue in verses and articles, and with the exception of a sonnet on "Peace" signed T. C. Chubb the most ambitious contribution in the February number is her "Desire of All Nations." Mr. Chubb, in a praiseworthy attempt to avoid banality, urges that after the present conflict a memory of war may be as tonic as the memory of peace is at present, but, perhaps too hastily, assumes that the nation was sunk in sloth before the war. Miss Nightingale treats the same theme as an allegory. A vision of peace comes to a statesman, a mother, the Emperor, and a dying soldier. To the first two she gives reasons why the struggle must be continued; to the Emperor no hope of her return to him; the soldier she gathers to herself. It is worth while to pith the allegory thus baldly, because when read as Miss Nightingale has written it the allegory makes a deeper impression. Her simple, direct, unaffected style has the virtue of its restraint, and when we compare her matter with her manner we can restate Buffon's famous definition of style by the phrase "style is the kernel of thought." "The matter is foreign to the man, and is not of him. The manner is the man himself." A gazette which excites reflections while provoking humour is as rare as it is refreshing.

### THE STATUS OF CHEMISTS.

The British Association of Chemists, which was founded in Manchester last November, has decided to form a new branch for the Leeds and Bradford districts. In the cause of unity an agreement is under consideration with the Institute of Chemistry, and Dr. Foster, who is one of the Association's organisers, argued that chemists needed to control their own affairs, from the teaching of chemistry to the making of finished products. Speaking as an industrial chemist, Mr. F. W. Richardson, county analyst, said that he sympathised with the aims of the Association, which should be composed of diverse sections, while the degree should be in the hands of the institute.

### THIS WEEK'S DRUG MARKET.

The shortage of American drugs, such as senega, cascara, and hamamelis, for instance, is becoming more pronounced, and high prices are asked for such supplies as are available. The noticeable falling-off in the arrivals of various synthetic drugs from America is also affecting prices; hexamine, for instance, is again dearer, as also are benzoic acid and formaldehyde. Extreme prices are paid for any sugar of milk offered; honey has advanced still further. Almond oil is dearer. The upward tendency in the price of bromides continues.

